# Association of Air Pollution Exposure with Incident Cataract Surgery and Neovascular Age-Related Macular Degeneration in 2 French Nationwide Cohorts

**DOI:** 10.1016/j.xops.2026.101099

**Published:** 2026-02-02

**Authors:** Laure Gayraud, Emeline Lequy, Emilie Hucteau, Kees de Hoogh, Mireille Coeuret-Pellicer, Cédric Schweitzer, Jean-François Korobelnik, Marie-Noelle Delyfer, Danielle Vienneau, Marcel Goldberg, Marie Zins, Cécile Delcourt

**Affiliations:** 1 University Bordeaux, INSERM, BPH, U1219, F-33000, Bordeaux, France; 2 Paris Cité University, “Population-based Cohorts Unit”, INSERM, Paris Saclay University, UVSQ, UMS 011, Paris, France; 3 Paris-Saclay University, UVSQ, Gustave Roussy, Inserm, U1018 CESP, Team “Exposome and Heredity”, F-94800, Villejuif, France; 4 Swiss Tropical and Public Health Institute, Allschwil, Switzerland; 5 University of Basel, Basel, Switzerland; 6 Institute for Risk Assessment Sciences (IRAS), Universiteit Utrecht, Utrecht, The Netherlands; 7 Centre Hospitalier Universitaire de Bordeaux, Service d'Ophtalmologie, Bordeaux, France; 8 Julius Center for Health Sciences and Primary Care, University Medical Center Utrecht, Utrecht, The Netherlands

**Keywords:** Air pollution, AMD, Cataract surgery, Cohort study, Eye diseases

## Abstract

**Purpose:**

Age-related macular degeneration (AMD) and cataracts are part of the leading causes of blindness globally, with oxidative stress being a key factor in their development. Air pollution, known to exacerbate oxidative stress, may contribute to the progression of these diseases. This study aims at investigating the association between ambient air pollution exposure and the incidence of cataract surgery and neovascular AMD (nAMD).

**Design:**

Two nationwide prospective French cohorts.

**Participants:**

The study involved 36 140 participants, including 17 911 Electricité de France-Gaz de France employees from the Gazel cohort and 18 229 individuals from the general French population in the Constances cohort, all aged over 53 years at inclusion and followed up for 10 years.

**Methods:**

Cataract surgery and nAMD cases were identified using the French National Health Data System (SNDS), which provided medical procedures and prescriptions. Air pollution exposure to nitrogen dioxide (NO_2_), fine particulate matter, and black carbon (BC) were estimated using land-use regression models. Cox proportional hazards models adjusted for age, sex, and education were used to assess associations. Analyses were stratified by area of residence (urban vs. rural), because of significant interactions.

**Main Outcome Measures:**

Neovascular AMD was identified through intravitreal anti-VEGF injections, and cataract surgery by the first surgery date, both extracted from SNDS data.

**Results:**

Among the 36 140 participants included (39% women, mean age 60.2 years), 5543 incident cataract surgery and 266 nAMD cases were identified. In the combined Constances and Gazel populations, urban individuals exposed to more than 34.8 μg/m^3^ of NO_2_ had an 8% higher risk of cataract surgery (HR = 1.08, 95% confidence interval [CI]: 1.01–1.16, *P* = 0.017), while no significant effect was observed among rural participants. Additionally, in the Constances cohort, participants exposed to the fourth quartile (≥2.33 10^-5^/m) of BC had an 88% increased risk of nAMD compared to those exposed to the first quartile (HR = 1.88, 95% CI: 1.03–3.45, *P* = 0.041). No significant association with BC was observed in the combined Constances and Gazel populations.

**Conclusions:**

This study provides new insights into the impact of air pollution on ocular aging at a national level, highlighting the distinct effects observed between urban and rural populations.

**Financial Disclosure(s):**

Proprietary or commercial disclosure may be found in the Footnotes and Disclosures at the end of this article.

Air pollution is responsible for millions of premature deaths each year and now represents the greatest threat to human health globally, surpassing tobacco and alcohol in its contribution to disease and mortality.^1^^,^^2^ It is involved in a wide range of health issues, from respiratory and cardiovascular diseases to cancer. More recently, a potential effect of air pollution on eye health has emerged, with some studies evidencing an association between exposure to certain air pollutants and age-related eye diseases such as cataracts and age-related macular degeneration (AMD).

Cataracts represent the main cause of blindness worldwide and AMD is a leading cause of irreversible vision loss among the elderly.^3^ Both conditions are major public health concerns due to their impact on quality of life and the significant burden they place on health care systems globally.^4^^,^^5^ The prevalence of cataracts and AMD increases significantly with age, affecting approximately 95 and 196 million of people worldwide, respectively.^5^^,^^6^

Cataracts are characterized by the progressive opacification of the lens, leading to a gradual decline in visual acuity and contrast sensitivity, ultimately impairing daily functioning.^5^ Age-related macular degeneration, on the other hand, represents the neurodegeneration of the macula, the central part of the retina, responsible for fine vision and essential for activities such as reading or face recognition.^7^ While cataract surgery is an effective treatment that can restore vision, it is associated with significant health care costs.^8^ In contrast, there is no curative treatment for AMD, although anti-VEGF injections can help slowing the progression of the neovascular form of the disease. This form is characterized by the abnormal growth of blood vessels under the retina. These fragile vessels can leak fluid or blood, leading to rapid and severe vision loss if untreated. When untreated, neovascular AMD (nAMD) accounts for the majority of severe vision loss,^9^ making the prevention and management of its risk factors particularly important.^4^

The etiology of both cataracts and AMD is complex and multifactorial, with oxidative stress being a well-established contributing factor in the development of both conditions.^10^^,^^11^ Chronic exposure to air pollutants, particularly gas such as nitrogen dioxide (NO_2_) and fine particulate matter (PM_2__.5_), with a diameter smaller than 2.5 μm and its component black carbon (BC), has been shown to exacerbate oxidative stress by increasing the production of reactive oxygen species, which can overwhelm the natural antioxidant defenses of the eye.^1^ In the lens, reactive oxygen species can lead to protein oxidation and aggregation, resulting in cataract formation, while in the retina, oxidative damage can contribute to the pathogenesis of AMD.^11^^,^^12^ Animal studies and in vitro trials have supported these mechanisms, demonstrating that oxidative stress can induce changes in the eye consistent with cataract and AMD development.^13^, ^14^, ^15^, ^16^

Despite the biological plausibility, epidemiological evidence linking air pollution exposure to cataracts and AMD remains limited and inconclusive. Only a few studies have investigated these associations, with mixed results and inconsistencies in the specific air pollutants implicated.^17^, ^18^, ^19^, ^20^, ^21^, ^22^, ^23^, ^24^, ^25^ Four studies from Canada, the United Kingdom, South Korea, and Taiwan have investigated the association between air pollution exposure and AMD.^17^, ^18^, ^19^, ^20^ Among them, only the Taiwanese study was longitudinal, and assessment of pollution exposure was crude, based on the pollution level at the nearest monitoring station^20^ rather than a spatial model of exposure at the participant's residences. This study found that participants in the highest quartile of NO_2_ exposure had an increased risk of AMD. These findings were supported by the cross-sectional study in South Korea^19^ but not by the UK Biobank study.^18^ Particulate matter was associated with AMD in both the Canadian study^17^ and the UK Biobank, but it was not assessed in the Taiwanese and South Korean studies.

Similarly, 5 studies have examined the link between cataracts and air pollution, only 3 being longitudinal.^17^^,^^22^, ^23^, ^24^, ^25^ Of these, only the UK Biobank study and our previous investigation in the Bordeaux Metropole population (3C-Alienor Study) specifically examined the association with cataract surgery. The UK Biobank reported an increased risk among participants with higher exposure to PM_2.5_ and NO_2_,^22^ whereas in the Bordeaux population, the association was observed only with NO_2_.^25^ Nitrogen dioxide was also positively associated with cataracts in the South Korean cross-sectional study^24^ but not in the Canadian study.^17^ In the studies that did not find an association, the outcome was either self-reported or based on the presence of cataracts rather than on cataract surgery, which could explain the differences. Additionally, the concentration ranges of pollutants varied significantly across these studies, with higher values in Asia and lower values in the United Kingdom or Canada. Finally, the relationship with BC was only explored in the UK Biobank and 3C-Alienor studies.

Given these gaps in the literature and the limitations of previous studies, our study aimed at further investigating the relationship between ambient air pollution exposure and the incidence of cataract surgery and nAMD in 2 nationwide prospective French cohorts.

## Methods

### Setting and Study Population

#### Gazel Cohort

The Gazel cohort (https://www.gazel.inserm.fr), established in 1989, consists of employees from the French energy production and distribution company “Electricité de France-Gaz de France”.^26^ The cohort included 20 625 participants, comprising at inception men aged 40 to 50 years and women aged 35 to 50 years, spread across France. At study entry, each participant completed a comprehensive self-administered questionnaire, collecting data on health status, lifestyle and socioeconomic factors. Participants have been followed up annually with similar questionnaires. In addition, data are regularly extracted from the Electricité de France-Gaz de France occupational health service records, and from 2007 to 2017, from the French National Health Data System (SNDS), which automatically records all medical and paramedical procedures for French residents. Residential histories for Gazel participants have been collected and geocoded since 1989. For this study, participants with at least 1 residential address provided, still alive and with data from the SNDS available in 2007 were selected, totaling 18 334 individuals.

The Gazel study protocol was approved by the French authority for data confidentiality (Commission Nationale de l'Informatique et des Libertés No. 105,728) and by the Ethics Evaluation Committee of the Institut National de la Santé et de la Recherche Médicale (Inserm, National Institute of Health and Medical Research) (IRB0000388, FWA00005831). The invitation to participate was sent by post to eligible persons, accompanied by a document detailing the project, the voluntary nature of their participation, the data collected, the conditions of security and confidentiality, and the future use of the data. The subjects solicited were invited to complete a questionnaire indicating their consent.

#### Constances Cohort

The Constances cohort (www.constances.fr) is a nationwide prospective cohort study of the general adult population in France, randomly selected from social security affiliates. The cohort includes approximately 220 000 adults, aged 18 to 69 at the time of inclusion, who were recruited between 2012 and 2019 to investigate a wide range of health determinants and diseases.^27^ Participants were recruited in 24 health screening centers located across France. At enrollment, participants completed detailed questionnaires covering their health and lifestyle, and underwent a standardized health examination. The follow-up involves annual self-administered questionnaires and periodic health examinations. Additionally, data from the SNDS are available in Constances, for the participants who gave their consent, from 2009 to 2021. Life-long residential histories for 80 600 participants of Constances have been collected and geocoded. Thus, 19 025 participants aged over 53 years at inclusion with at least 1 provided residential address were considered in this study, as this age threshold was chosen to ensure comparability with the Gazel cohort.

In agreement with French regulations, the Constances study was authorized by the National Data Protection Authority (Commission Nationale de l'Informatique et des Libertés, CNIL-#910486) and approved by the Institution Review Board of the National Institute for Medical Research (INSERM, #01-011). All participants signed a written informed consent.

Both cohorts were established by the same research team, enabling the harmonization of methodologies and collected variables, which facilitated the conduct of pooled analyses.

### Cataract Surgery and Neovascular AMD Assessment

The linkage of the Constances and Gazel cohorts with the national medico-administrative databases (Système National de Données de Santé [SNDS]) provides access to health care expenditures reimbursed by the national health insurance system, including medications and medical procedures. It also includes demographic data (age, sex, vital status) and diagnoses of long-term illnesses (Affection de Longue Durée), coded according to the International Classification of Diseases 10, as well as patient stays in public and private hospitals, discharge diagnoses, and medical procedures performed during hospital stays. These SNDS data allow identifying cases of nAMD across all regions of France, allowing a comprehensive tracking of the care pathway, including ophthalmological care and specific treatments for nAMD, as well as cataract surgery procedures.^28^, ^29^, ^30^ For the present study, the study period was 1 January 2007 to 31 December 2017 for all participants of the Gazel cohort and 1 January 2012 to 31 December 2021 for all participants of the Constances cohort.

### Cataract Surgery

Incident cataract surgery was identified through linkage with SNDS procedure records. Participants were classified as having undergone cataract surgery if they had a Classification Commune des Actes Médicaux medical procedure code of BFGA004 (“Extracapsular extraction of the lens by phacoemulsification”) before 2019, or the codes BFGA427 (“Extracapsular extraction of the lens by phacoemulsification, with implantation of an artificial lens in the posterior chamber of the eye, without trabecular drainage device implantation”) or BFGA368 (“Extracapsular extraction of the lens by phacoemulsification, with implantation of an artificial lens in the posterior chamber of the eye, with trabecular drainage device implantation ab interno”) from 2019 onward. The incidence of cataract surgery was defined as the first cataract surgery performed between 2012 and 2021 for the Constances cohort, and between 2007 and 2017 for the Gazel cohort.

### Neovascular AMD

Neovascular AMD cases were identified from SNDS data using an algorithm that combined the identification of anti-VEGF prescriptions (ranibizumab, aflibercept, bevacizumab, and pegaptanib; Anatomical Therapeutic Chemical codes: S01LA03, S01LA04, S01LA05) with the detection of nAMD diagnoses within the SNDS. This algorithm excluded other retinal conditions that could serve as differential diagnoses, such as vein occlusion and diabetic macular edema (see Fig S1, available at www.ophthalmologyscience.org).

The incidence of nAMD was defined as the date of the first anti-VEGF prescription identified between 2012 and 2021 for the Constances cohort, and between 2007 and 2017 for the Gazel cohort.

### Air Pollutant Exposure Assessment

As part of the ELAPSE (Effects of Low-Level Air Pollution: A Study in Europe) initiative, detailed air pollution maps at a 100 × 100 m resolution were created for Western Europe, representing annual average concentrations of PM_2.5_ (μg/m^3^), BC (10^–5^/m), and NO_2_ (μg/m^3^) for 2010. The Land Use Regression models were developed using measurements from 2399 NO_2_ and 543 PM_2.5_ routine monitoring stations from the European Environment Agency's Airbase network, and 436 BC monitoring locations derived from the European Study of Cohorts for Air Pollution Effects project.^31^ Predictor variables included land-use characteristics, proximity to major roads, elevation, distance from the coastline, and population density, as well as data from chemical transport models and satellite observations.^32^^,^^33^ These Land Use Regression models explained 66%, 51%, and 58% of the spatial variation in PM_2.5_, BC, and NO_2_ concentrations, respectively. All available geocoded addresses were linked to the 2010 maps; then these estimates were rescaled annually to cover the years 1990 to 2019 by incorporating temporal air pollution data and regional variations. Residential histories for each participant of each cohort have been collected and geocoded since 1989. For the present study, for each individual and pollutant, exposure levels were estimated and assigned as the 10-year average prior to the start of the study period (i.e., 1997–2007 for all participants of Gazel and 2002–2012 for all participants of Constances).

### Covariates

Covariates used in the multivariable analyses were collected at baseline and identified as potential confounders using a Directed Acyclic Graph (Figs S2 and S3, available at www.ophthalmologyscience.org). Only age, sex, and level of education (categorized as low: primary and middle school diploma; intermediate: professional certification; and high: high school diploma, university degree, or advanced technical certification) were identified as potential confounders and were adjusted for in the main analyses. Participants living in towns with fewer than 20 000 residents at the time of inclusion were classified as residing in rural areas. The variables used for the descriptive analysis were defined as follows. Comorbidities were identified using the national insurance system (SNDS) and included diabetes (defined as a long-term condition for diabetes, classification according to International Classification of Diseases 10, or use of antidiabetic medication), hypertension (use of antihypertensive drugs), and oral corticosteroid therapy (use of corticosteroid drugs at least twice per year on average over 3 years). Body mass index, calculated as weight (kg)/height (m),^2^ was categorized into 3 groups (<25; [25–30]; >30) as defined by the World Health Organization. Smoking status was self-reported and classified as never smoked, <20 pack-years, or ≥20 pack-years. Adherence to the Mediterranean diet (MeDi) was assessed using the MeDi score developed by Sofi et al^34^ and was used as a quantitative variable in the analyses.

### Statistical Analysis

First, the population included in this analysis (>53 years old) was compared to the overall populations >53 years of the Constances and Gazel cohorts. Next, the associations between exposure to NO_2_, PM_2.5_, and BC with cataract surgery and nAMD incidence were evaluated using Cox proportional hazard models. The time axis was defined as the time from baseline (January 1, 2007 for Gazel, January 1, 2012 for Constances) to either the date of the first cataract surgery or the last year of available SNDS data for cataracts, and from baseline to the first anti-VEGF prescription or the last year of available SNDS data for nAMD. All models were single-pollutant, because of high correlations between pollutants.

The models were adjusted for age at inclusion, sex, and educational level (for both nAMD and cataract), and additionally for study cohort (Constances vs. Gazel) in the overall analyses, based on potential confounders identified using a Directed Acyclic Graph (Figs S2 and S3). Interactions between air pollution exposure and smoking status, sex, and type of residential area were tested. Only the interaction with residential area was statistically significant in some models. Therefore, for consistency and comparability across analyses, results were systematically presented separately for urban and rural areas in all models. For nAMD, the number of cases was too low in rural areas and the results were presented for the overall population only.

The linearity of quantitative variables was checked using 3-node restricted cubic splines, and the proportionality of hazards was assessed with the Schoenfeld test. Since linearity was adequate, the results for the pollutant parameters were expressed per interquartile range (IQR) increment. After examining the progression of cataract risk relative to NO_2_ concentration, a threshold of 34.8 μg/m^3^ was identified, beyond which a gradual increase in risk was observed. The variable was then dichotomized at this threshold to compare the risk between participants exposed to more than 38.4 μg/m^3^ and those exposed to lower levels. As a clear threshold was not identified for BC, the variable was categorized into quartiles in order to compare the risk among participants most exposed (fourth quartile) to those least exposed (first quartile). Additional sensitivity analyses were conducted using extended adjustment models that included established risk factors for the outcomes beyond traditional confounders. For cataract surgery, models were additionally adjusted for smoking status, body mass index categories, diabetes, hypertension, and corticosteroid use. For nAMD models were additionally adjusted for smoking status, adherence to the MeDi (MEDI-LITE score), and hypertension. A sensitivity analysis was also conducted after excluding participants with less than 80% of available pollution data over the 10-year exposure window. These analyses are presented in the Supplementary Material. All statistical analyses were performed using R, version 2023.06.2 (R Core Team) with the package Survival.

## Results

### Descriptive Analysis

**Figure 1:**
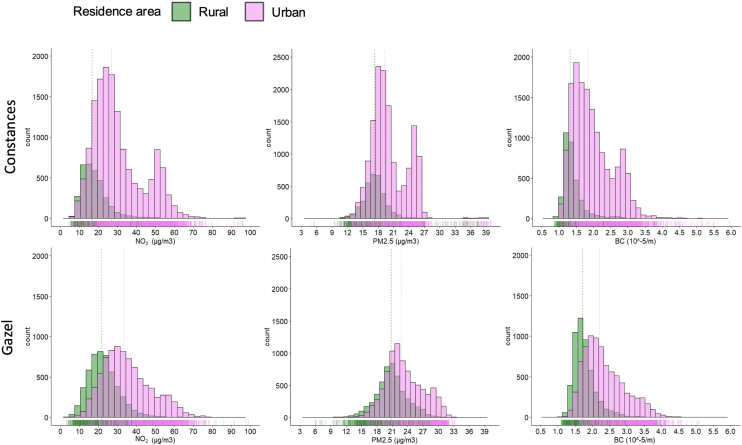
Histograms: distribution of NO_2_, PM_2.5_, and BC in the Constances and Gazel cohorts. BC = black carbon; NO_2_ = nitrogen dioxide; PM_2.5_ = particulate matter.

Of the 37 359 participants aged more than 53 years in the Gazel (N = 18 334) and Constances (N = 19 025) cohorts, 36 140 were included in the analyses. Among the nonincluded participants, 94 from the Gazel cohort had died before 2007, and 253 participants from the Constances and 8 from the Gazel cohorts had undergone cataract surgery or had prevalent nAMD at baseline (244 with cataract surgery and 9 with nAMD for Constances and 8 cataract surgery for Gazel). Additionally, 864 participants had missing data for air pollution exposure (321 from Gazel and 543 from Constances) (comparison overall vs. included Table S1, available at www.ophthalmologyscience.org). Among the 36 140 included participants, 39% were women and the mean age at baseline was 60.2 years (standard deviation: 4.6) (Table 1). Overall, 48% of the sample had an intermediate level of education, 73% lived in urban areas, 66% had never smoked, 13% had a body mass index of 30 kg/m^2^ or more, 36% had a cardiovascular disease, 8% had diabetes, and 11% were hypertensive. The median (IQR) individual exposures estimated at the participants' residential addresses over the 10 years preceding baseline were 26.28 (16.99) μg/m^3^ for NO_2_, 20.39 (5.55) μg/m^3^ for PM_2.5_, and 1.85 (0.80) 10^-5^/m for BC (Table 1; Fig 1. histograms of the distribution). During the 10 years of follow-up, a total of 5543 incident cataracts (2202 in Constances and 3341 in Gazel) and 266 nAMD cases (107 in Constances and 159 in Gazel) were identified. When comparing the 2 cohort populations, participants from the Gazel cohort had a lower percentage of women (28% vs. 49%), a lower proportion with a high level of education, and were more often rural residents. They were also less likely to be smokers, more adherent to the MeDi, and had lower rates of hypertension and cardiovascular diseases. Overall, participants who developed nAMD or underwent cataract surgery during the follow-up period were older. Those with nAMD had lower levels of education, were more likely to be heavy smokers, and had higher rates of hypertension. Participants who underwent cataract surgery during the follow-up had similar education levels to those who did not undergo surgery but were more frequently affected by diabetes and cardiovascular diseases, and were more likely to receive corticosteroid treatment. Overall, participants who had cataract surgery or nAMD during the follow-up had received more exposure to air pollution compared to the general population.

**Table 1:** Baseline Characteristics of the Study Population

Characteristic	Overall	Constances	Gazel	Overall	
	N = 36 140 N (%)	N = 18 229 N (%)	N = 17 911 N (%)	Cataract Surgery, N = 5543 N (%)	nAMD, N = 266 N (%)
Women	13 944 (39)	9002 (49)	4942 (28)	2160 (39)	96 (36)
Age (mean [SD])	60.16 (4.59)	58.17 (4.68)	62.18 (3.49)	62.51 (3.88)	62.74 (3.98)
Education level					
Low	5467 (15)	1667 (9)	3800 (22)	1014 (19)	53 (20)
Intermediate	17 171 (48)	6730 (38)	10 441 (60)	2667 (49)	132 (51)
High	12 366 (35)	9474 (53)	2892 (16)	1668 (31)	73 (28)
Other	456 (1)	51 (0.3)	405 (2.3)	79 (1)	2 (0.8)
Unknown	680	307	373	115	6
Residence area					
Rural	8443 (27)	3329 (18)	5114 (38)	1323 (28)	79 (34)
Urban	23 281 (73)	14 893 (82)	8388 (62)	3401 (72)	155 (66)
Unknown	4416	7	4409	819	32
Smoking					
Never smoker	22 419 (66)	7779 (46)	14 640 (85)	3642 (70)	169 (68)
<20 pack/years	8786 (26)	6523 (39)	2263 (13)	1180 (23)	50 (20)
≥pack/years	2710 (8.0)	2487 (15)	223 (1)	396 (8)	30 (12)
Unknown	2225	1440	785	325	17
BMI					
(0,25)	15 567 (46)	8958 (50)	6609 (42)	2227 (44)	103 (42)
(25,30)	13 654 (41)	6675 (37)	6979 (44)	2125 (42)	106 (43)
≥30	4488 (13)	2355 (13)	2133 (14)	760 (15)	37 (15)
Unknown	2431	241	2,19	431	20
Mediterranean diet adherence, median (IQR) range	9.60 (3.00) 1.20–17.40	8.80 (3.00) 1.20–17.00	10.20 (2.70) 4.00–17.40	9.80 (3.00) 2.90–17.30	10.00 (2.98) 5.00–16.20
Diabetes	2709 (7.5)	1155 (6.3)	1554 (8.7)	593 (11)	26 (9.8)
Hypertension	3973 (11)	3189 (17)	784 (4.4)	714 (13)	42 (16)
Cardiovascular diseases	13 058 (36)	7626 (42)	5432 (30)	2285 (41)	114 (43)
Corticosteroid therapy	5187 (14)	2466 (14)	2721 (15)	947 (17)	41 (15)
NO_2_ μg/m^3^ (median [IQR] min-max)	26.28 (16.99) 3.02–125.39	24.84 (15.38) 4.13–125.3 9	28.04 (17.30) 3.02–100.39	27.11 (17.79) 3.14–93.98	27.26 (18.10) 5.32–74.67
PM_2.5_ μg/m^3^ (median [IQR] min-max)	20.39 (5.55) 5.01–52.43	18.87 (4.13) 5.01–52.43	21.84 (4.71) 5.61–33.71	20.84 (5.42) 5.93–49.67	20.84 (4.62) 13.35–35.50
BC 10^-5^/m (median [IQR] min-max)	1.85 (0.80) 0.51–7.06	1.71 (0.80) 0.51–7.06	1.97 (0.77) 1.09–5.15	1.91 (0.82) 0.89–5.94	1.92 (0.75) 1.08–4.16

BC = black carbon; BMI = body mass index; IQR = interquartile range; nAMD = neovascular AMD; NO_2_ = nitrogen dioxide; PM_2.5_ = particulate matter; SD = standard deviation; min-max: minimum-maximum.

### Multivariable Analysis

The association of cataract surgery risk with varying concentrations of air pollutants is illustrated using Cox modeling with cubic splines, adjusting for age, sex, and education level (Figs S4–S6, available at www.ophthalmologyscience.org). The hazard ratio for cataract surgery increased with NO_2_ and BC exposure starting from 34.8 μg/m^3^ for NO_2_ (Fig S4) and 2.33 10^–5^/m for BC (Fig S6) (the cutoff value of the fourth quartile for this pollutant) among urban participants in the Constances cohort. No significant effect was observed for PM_2.5_ (Fig S5) or for any pollutant in the Gazel cohort or among rural participants in the Constances cohort.

For nAMD in the Constances population, a slight and gradual increase in the risk was observed starting at 2.33 10^–5^/m of BC (fourth quartile) (Fig S6). No other significant variations in the risk of nAMD were found in relation to air pollution exposure.

**Figure 2:**
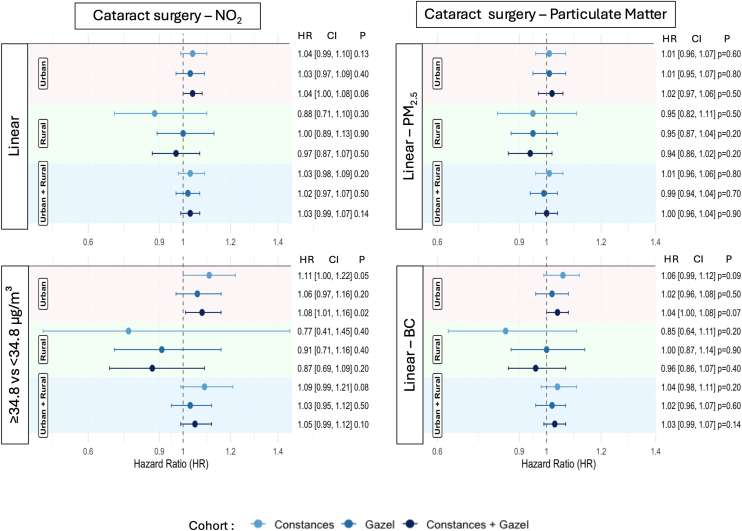
Forest plot of associations between air pollution exposure and risk of cataract. BC = black carbon; CI = confidence interval; NO_2_ = nitrogen dioxide; PM_2.5_ = particulate matter.

**Figure 3:**
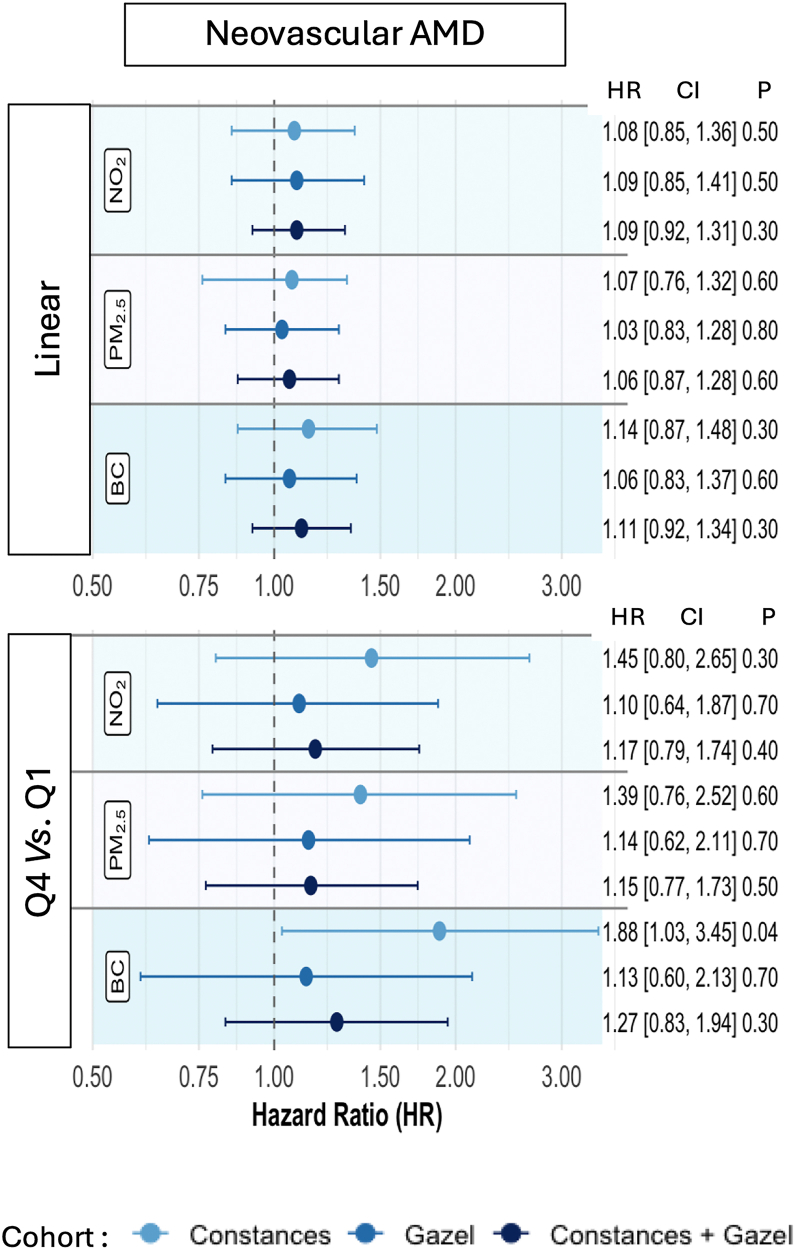
Forest plot of associations between air pollution exposure and risk of nAMD. AMD = age-related macular degeneration; BC = black carbon; CI = confidence interval; NO_2_ = nitrogen dioxide; PM_2.5_ = particulate matter.

The results of the Cox models fits are presented in the forest plots (Figs 2 and 3). Urban participants in the Constances and Gazel cohorts exposed to more than 34.8 μg/m^3^ of NO_2_ showed a 11% (confidence interval [CI]: 1.00–1.22, *P* = 0.05) and 6% (CI: 0.97–1.16, *P* = 0.20) increase in hazard ratio (HR) for incident cataract surgery compared to those exposed to less than 34.8 μg/m^3^, respectively, although both of these associations were not statistically significant (Fig 2). In the combined population (Constances and Gazel), urban participants exposed to more than 34.8 μg/m^3^ of NO_2_ had a significant 8% increase in incident cataract surgery risk (CI: 1.01–1.16, *P* = 0.017), compared to those exposed to less than 34.8 μg/m^3^. No significant association of NO_2_ with incident cataract surgery was found among rural participants or with PM_2.5_ and BC exposure.

For nAMD, an association with BC was observed in the Constances cohort (Fig 3), where participants exposed to the fourth quartile of BC had an 88% increased risk (CI: 1.03–3.45, *P* = 0.041) of developing nAMD during the follow-up compared to those in the first quartile (Fig 3). This association was not observed in the Gazel cohort or in the combined (Gazel and Constances) population. Due to the limited number of nAMD cases, it was not possible to explore potential differences between rural and urban participants.

### Sensitivity Analyses

#### Extended Adjustment for Risk Factors

In sensitivity analyses with extended adjustment for established risk factors of the outcome, beyond traditional confounders, effect estimates were attenuated compared with the main models. For cataract, results were very similar (in particular, HR = 1.07; 95% CI: 1.00-1.14; *P* = 0.05), although at the limit of statistical significance (Fig S7, available at www.ophthalmologyscience.org). Regarding nAMD, the results were somewhat attenuated (HR = 1.48; 95% CI: 0.83-2.64; *P* = 0.18) (Fig S8, available at www.ophthalmologyscience.org).

#### Restriction to Participants with High Exposure Data Completeness

After excluding participants with less than 80% of available pollution data over the 10-year exposure period (N = 278), results for cataract surgery were similar to those observed in the main analysis (Tables S2 and S3, available at www.ophthalmologyscience.org). For nAMD, the trend of the association with BC remained consistent, although *P* values were borderline and no longer statistically significant (Table S4, available at www.ophthalmologyscience.org).

## Discussion

In the overall population (Gazel and Constances), urban participants exposed to NO_2_ levels above 34.8 μg/m^3^ had an 8% increased risk of cataract surgery (CI: 1.01–1.16, *P* = 0.017) compared to those exposed to lower levels, with no similar association observed among rural residents. No effect was found for exposure to PM_2.5_ and BC and cataract surgery.

For nAMD, in the Constances population (urban and rural), participants exposed to the fourth quartile of BC had an 88% increased risk of developing AMD (HR: 1.88; CI: 1.03–3.45; *P* = 0.041) compared to those in the first quartile. This association was not observed in the Gazel cohort or in the combined Constances and Gazel population. However, in sensitivity analyses including additional adjustment for established risk factors of cataract and nAMD, effect estimates were attenuated and the association was no longer statistically significant.

For cataract, we found that urban participants exposed to NO_2_ levels above 34.8 μg/m^3^ had an 8% increased risk of cataract surgery (CI: 1.01–1.16, *P* = 0.017) compared to those exposed to lower levels. Further adjustment for established cataract risk factors in sensitivity analyses yielded similar effect estimates, although the associations were no longer statistically significant. This attenuation suggests that the observed association may be partly influenced by the inclusion of additional risk factors related to cataract development, some of which may potentially act as mediators in the relationship between air pollution exposure and the outcome, particularly metabolic factors.^35^ This finding aligns with results from the South Korea study, where participants in the highest quartile of NO_2_ exposure also had an 8% higher hazard ratio compared to those in the lowest quartile,^24^ and consistent with the UK Biobank study, which reported a 4% increased risk per IQR increment,^22^ and with the 3C-Alienor study, where exposure to concentrations ≥40 μg/m^3^ of NO2 was associated with a 46% higher risk.^25^ The 2 other studies exploring the effect of air pollution on cataracts did not examine the associations with NO_2_.^17^^,^^23^

We did not find any statistically significant association of PM_2.5_ exposure with the incidence of cataracts. These results are consistent with findings from Canadian and Korean studies.^17^^,^^24^ However, in the UK Biobank, each IQR increase in PM_2.5_ was associated with a 5% higher hazard ratio for developing cataracts.^22^ This difference could be due to the varying composition of PM_2.5_ across different geographic regions. This hypothesis is supported by the similarity in NO_2_ and PM_2.5_ findings in the UK study, suggesting that the correlation between PM_2.5_ and NO_2_ might be stronger in the UK Biobank than in our study. This suggests that the observed effect of PM_2.5_ could potentially reflect the effect of NO_2_ in the UK Biobank. Indeed, the correlation between PM_2.5_ and NO_2_ can vary depending according to their primary sources. Additionally, the UK Biobank only considered exposure to PM_2.5_ in 2010, without extrapolation to other years, which limits the exploration of the air pollution effects and increases the risk of exposure misclassification. Finally, the exposure range in the UK study was much lower than that observed in our national cohort studies. A “plateau effect” at higher exposure levels cannot be ruled out.

We did not find any association of BC with cataract surgery.^22^ In the UK biobank analysis, an IQR increase in BC was associated to an 8% higher risk of cataract in a model adjusted for sex and age. However, this effect was no longer observed after more adjustments.

We observed that the impact of pollution was significant only among urban residents for cataract surgery, suggesting an interaction with urban versus rural settings. The differences in results between urban and rural areas may be explained by variations in pollution levels, as well as differences in pollution sources, with potentially lower exposure to traffic-related emissions in rural regions. To the best of our knowledge, this is the first study to explore this specific interaction. The UK Biobank, which is methodologically the closest study to ours and shows similar pollution effects overall, aligns with our findings for urban participants. This coherence is likely because 85% of the UK Biobank population resides in urban areas.^36^ For the Korean studies, which had a similar proportion of rural participants to our study,^37^ an effect of NO_2_ was observed in the overall population without distinguishing between urban and rural areas.^24^ However, the average concentrations and the exposure range were significantly higher than in our study.

We did not find any significant association of NO_2_ with incident nAMD. The small number of cases may have led to a lack of statistical power. In the Korean cross-sectional study^19^ and the Taiwanese longitudinal study,^20^ exposure to high NO_2_ concentrations was associated with AMD, whereas this was not observed in the UK Biobank cross-sectional study.^18^ However, in the Asian studies, the concentration of NO_2_ was much higher than in Europe.

We did not observe a significant association between PM_2.5_ exposure and nAMD in our study. In contrast, cross-sectional studies conducted in Canada and the UK Biobank reported associations between higher PM_2.5_ exposure and AMD. These associations were not evaluated in the Asian studies. However, in the Constances population (urban and rural), participants exposed to the fourth quartile of BC had an 88% increased risk of developing AMD (HR: 1.88; CI: 1.03–3.45; *P* = 0.041) compared to those in the first quartile. This association was not statistically significant in sensitivity analyses with extended adjustment for established risk factors of nAMD. In previous research, only the UK Biobank study examined the association of BC with AMD, and they did not find a significant association.^38^ Notably, that study was cross-sectional, and the BC concentrations were lower than in our study (median [IQR] 1.22 [0.33], range 0.83–4.05 in the UK Biobank vs. 1.85 [0.80], range 0.51–7.06 in our study).

The consistency of these findings with biological mechanisms observed in animal models strengthens their plausibility.^14^, ^15^, ^16^ Inhalation of NO_2_ and PM_2.5_ triggers inflammatory responses, leading to cytokine production and oxidative stress through reactive oxygen species. Oxidative stress is a well-established mechanism in the development of both cataracts and AMD.^10^, ^11^, ^12^, ^13^ Additionally, due to their small diameter, fine PM_2.5_ can penetrate deep into the body, crossing blood vessels and potentially the blood-retinal barrier, thereby reaching the retina and activating immune cells (microglia), leading to further inflammation and oxidative stress. While these biological mechanisms are compatible with a potential role of air pollution, the attenuation of associations observed after adjustment for established risk factors should be interpreted cautiously. In the absence of a formal mediation analysis, these results do not allow disentangling direct and indirect effects, but rather suggest that part of the observed associations may be sensitive to the inclusion of additional factors related to disease development, such as cardiometabolic conditions and systemic inflammation.

The strengths of our study include a large national sample size with an extended follow-up period of 10 years. Exposure to air pollution was estimated using Land Use Regression modeling rescaled annually and at individual home addresses, which enabled the estimation of annual individual pollution concentrations over a long-term follow-up period. Additionally, the inclusion of residential address history allowed for regular updates of exposure data. The use of data from the SNDS to estimate ocular pathologies further strengthens the study, as it ensures reliable diagnostics and precise dates for estimating time-to-event intervals. Moreover, the diverse population of our cohorts allowed us to study and highlight urban–rural differences, a distinction that had not been previously investigated.

The study has several limitations. The small number of nAMD cases limits the statistical power. In particular, we could not estimate associations of air pollutants with nAMD in rural participants. Additionally, we were only able to study nAMD due to the lack of reimbursed treatments for atrophic AMD in France, which prevented the identification of specific treatments in the SNDS. Moreover, a small number of participants may have had prevalent nAMD at baseline, as information prior to 2007 was not available; however, given the relatively young age of the study population at inclusion, the number of such cases is expected to be low. Furthermore, cases of nAMD were identified using an algorithm based on anti-VEGF injections, while excluding differential diagnoses, which reduces but does not completely eliminate the possibility of misclassification. Cataract was defined based on cataract surgery rather than a diagnosis of cataract, likely capturing only more advanced cases. Air pollution exposure was estimated rather than directly measured, using land-use regression models, which may introduce some exposure estimation error. Additionally, there was no data on indoor pollution, real-time pollution, and occupational exposure for the participants, and we lacked information on ultraviolet exposure, a known risk factor for cataracts. We were also unable to account for ethnicity, as the collection of such data is restricted in France, although it is likely that the majority of the samples were of European descent. Finally, the differences in findings between Constances and Gazel may be explained by the specific nature of the Gazel cohort, which represents a professional population and is less representative of the general population.

## Conclusion

This study provides insights into the relationship between air pollution and ocular aging. Our findings suggest that exposure to higher level of NO_2_ is associated with an increased risk of cataract surgery, particularly among urban residents, highlighting the importance of considering urban–rural differences in environmental health research. Exposure to higher level of BC was associated with an increased risk of incident nAMD in the Constances cohort, but not in the overall population. However, the limited number of cases may have affected the statistical power of the study regarding nAMD. Overall, our results support a potential role of air pollution in ocular aging; however, further studies are needed to better characterize this relationship and to clarify the underlying mechanisms involved.
