# Supplementary material for: Association of Air Pollution Exposure with Incident Cataract Surgery and Neovascular Age-Related Macular Degeneration in 2 French Nationwide Cohorts
Source: Ophthalmol Sci. 2026 Feb 2;6(4):101099. doi: 10.1016/j.xops.2026.101099 (PMC12993142; doi:10.1016/j.xops.2026.101099)
Supplement: Figures S1–S8 and Tables S1–S4 [file mmc1.pdf]

Association of Air Pollution with Incident Cataract Surgery and Neovascular Age-Related Macular Degeneration in Two French Nationwide Cohorts

Laure Gayraud[1], Emeline Lequy[2], Emilie Hucteau[1], Kees de Hoogh[3,4], Mireille Coeuret-Pellicer[2], Cédric Schweitzer[1,5], Jean-François Korobelnik[1,5], Marie-Noelle Delyfer[1,5], Danielle Vienneau[3,4], Marcel Goldberg[2], Marie Zins[2,6], Cécile Delcourt[1].

1. Univ. Bordeaux, INSERM, BPH, U1219, F-33000 Bordeaux, France
2. Univ. Paris Descartes, INSERM, Population-Based Epidemiological Cohorts, UMS 011, F-94807, Villejuif, France
3. Swiss Tropical and Public Health Institute, Allschwil, Switzerland
4. University of Basel, Basel, Switzerland
5. Centre Hospitalier Universitaire de Bordeaux, Service d'Ophtalmologie, Bordeaux, France
6. Paris-Saclay University , Gif-sur-Yvette , France.

SUPPLEMENTARY MATERIAL

Figure S1. Algorithm for identifying cases of neovascular AMD (nAMD) 3
Figure S2. Directed acyclic graph illustrating the association between air pollution and cataract 4
Figure S3. Directed acyclic graph illustrating the association between air pollution and age-related macular degeneration 5
Table S1. Comparison of overall and included population 6
Figure S4. Evolution of cataract surgery and neoascular age-related macular degeneration (nAMD) risk according to NO_2 concentration estimated using splines 7
Figure S5. Evolution of cataract surgery and neoascular age-related macular degeneration (nAMD) risk according to $PM_{2.5}$ concentration estimated using splines 8
Figure S6. Evolution of cataract surgery and neoascular age-related macular degeneration (nAMD) risk according to BC concentration estimated using splines 9
Sensitivity analysis 10
Sensitivity Analysis 1: Extended Adjustment Including Outcome Risk Factors 10
Figure S7. Forest plot of associations between air pollution exposure and risk of cataract (Model 2) 11
Figure S8. Forest plot of associations between air pollution exposure and risk of nAMD (Model 2) 12

Sensitivity Analysis 2: Restriction to Participants with High Exposure Data Completeness 13
Table S2. Baseline characteristics of the study population 13
Table S3: Association of Air Pollution with Incidence of cataract Surgery estimated by Cox Models 15
Table S4: Association of Air Pollution with Incidence of Neovascular AMD Estimated by Cox Models 16
Table S3: Association of Air Pollution with Incidence of cataract Surgery estimated by Cox Models 17

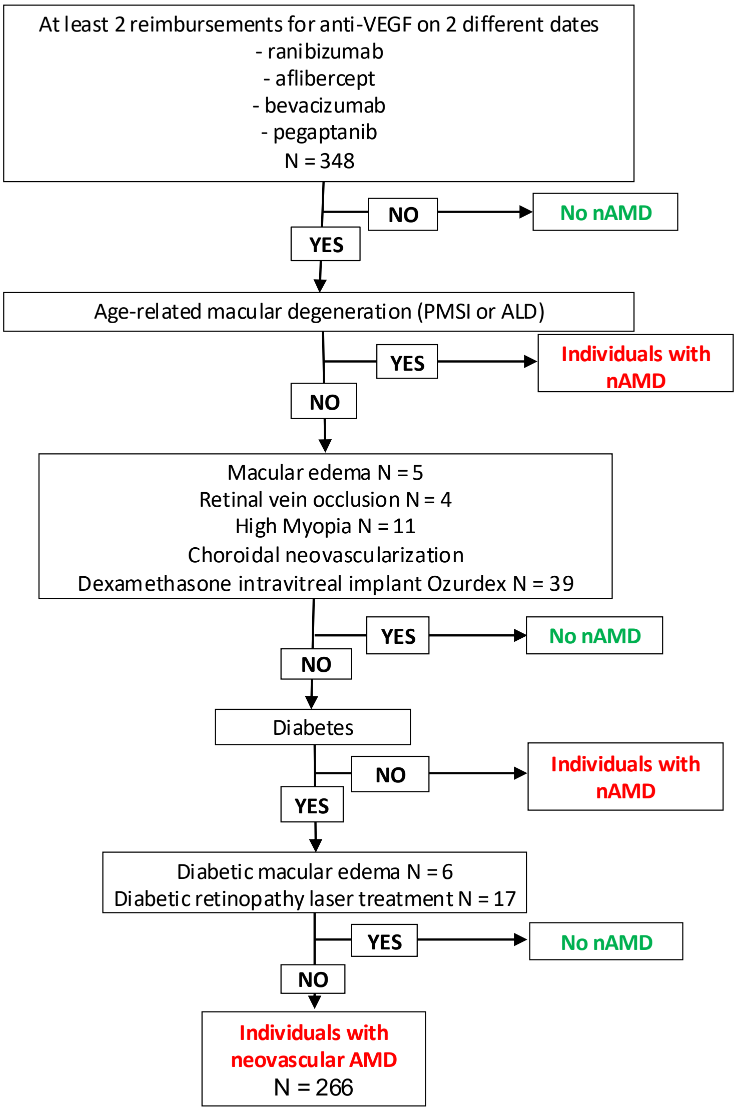

Figure S1. Algorithm for identifying cases of neovascular AMD (nAMD)

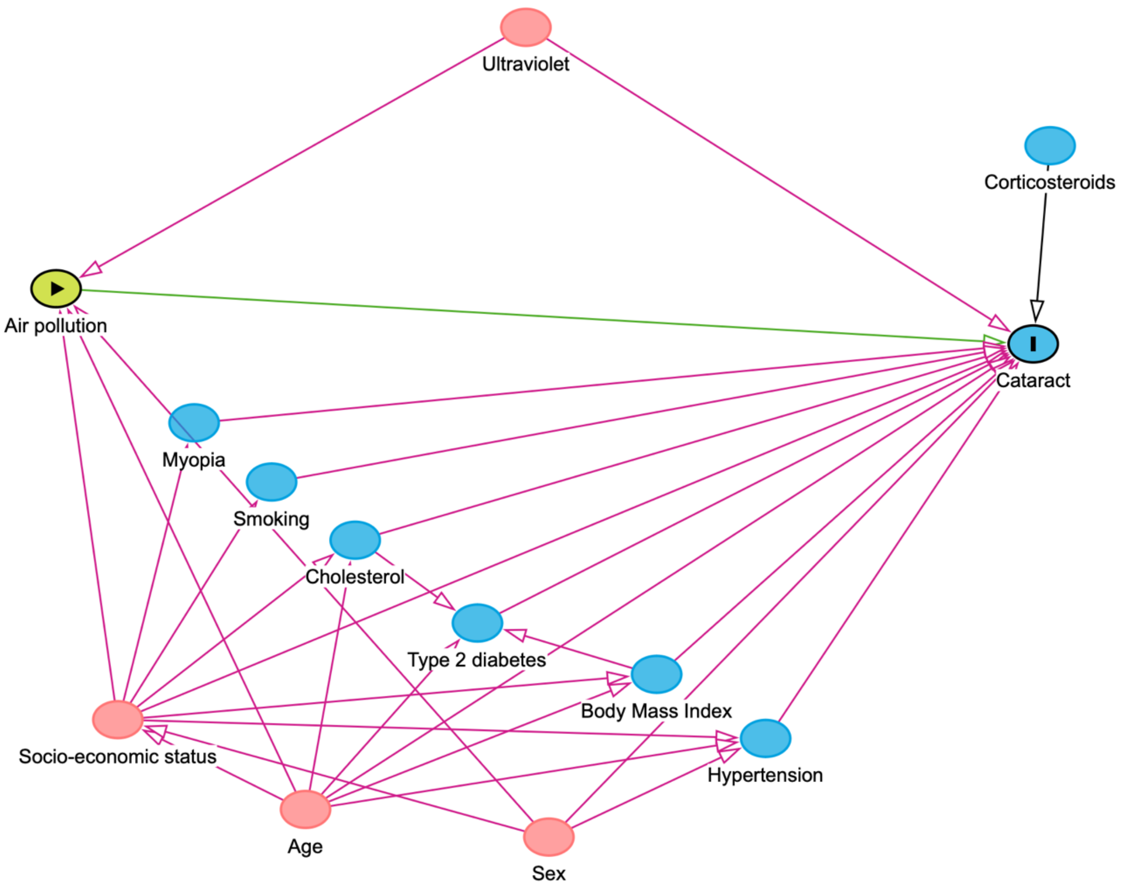

Figure S2. Directed acyclic graph illustrating the association between air pollution and cataract

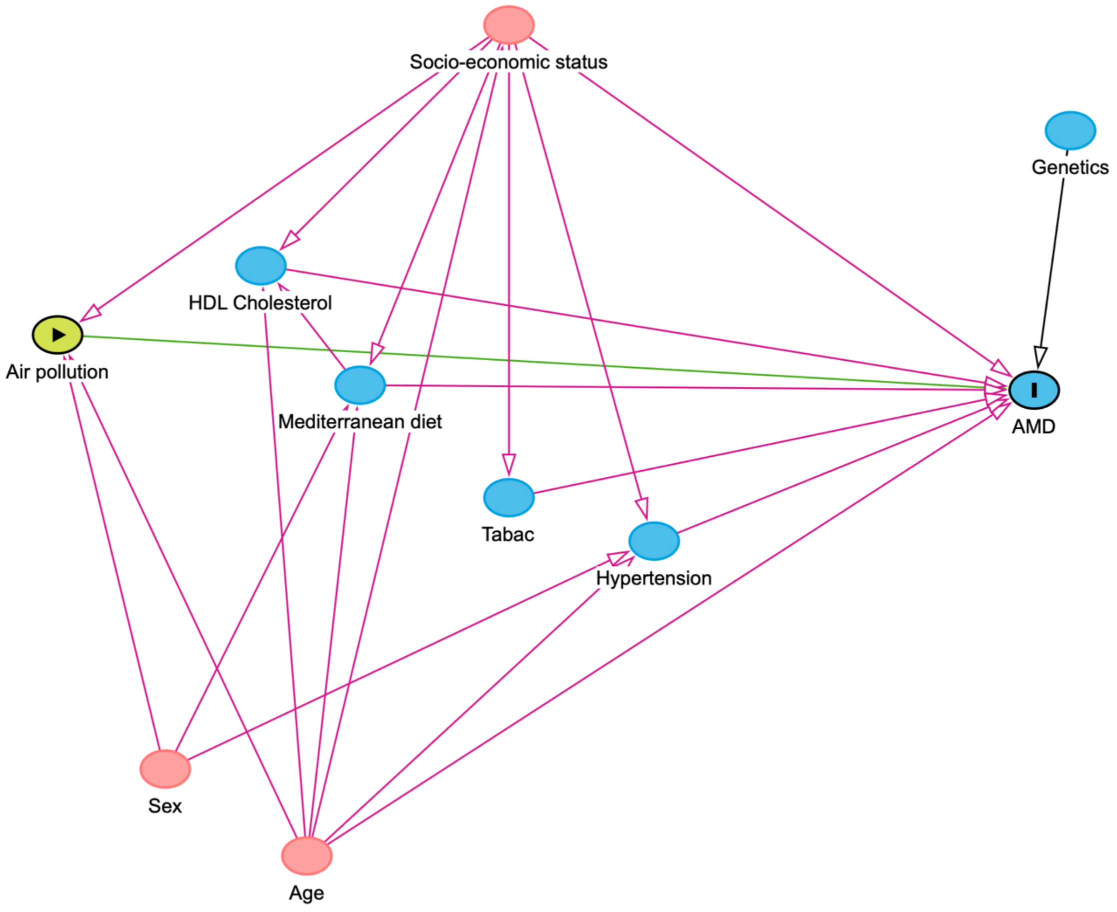

Figure S3. Directed acyclic graph illustrating the association between air pollution and age-related macular degeneration

Table S1. Comparison of overall and included population

Characteristic	Overall, N = 37,409<br>N (%)	Included, N = 36,140<br>N (%)
**Women**	14,498(39)	13,944(39)
**Age (Mean (SD))Min-Max**	62.35(3.94) 54.00 - 73.50	62.33(3.93) 54.00 - 73.50
**Education level**		
low	12,836(35)	12,366(35)
intermediate	17,722(48)	17,171(48)
high	5,672(15)	5,467(15)
other	471(1.3)	456(1.3)
Unknown	708	680
**Residence area**		
Rural	8,675(26)	8,443(27)
Urban	24,063(74)	23,281(73)
Unknown	4,671	4,416
**Smoking**		
No smoking	23,126(66)	22,419(66)
<20 pack/years	9,040(26)	8,786(26)
>=20 pack/years	2,926(8.3)	2,710(8.0)
Unknown	2,317	2,225
**BMI**		
[0,25)	16,100(46)	15,567(46)
[25,30)	14,106(40)	13,654(41)
[30,100]	4,629(13)	4,488(13)
Unknown	2,574	2,431
**Diabetes**	2,810(7.5)	2,709(7.5)
**Hypertension**	4,157(11)	3,973(11)
**Cardiovascular diseases**	13,560(36)	13,058(36)

Figure S4. Evolution of cataract surgery and neoascular age-related macular degeneration (nAMD) risk according to NO_2 concentration estimated using splines

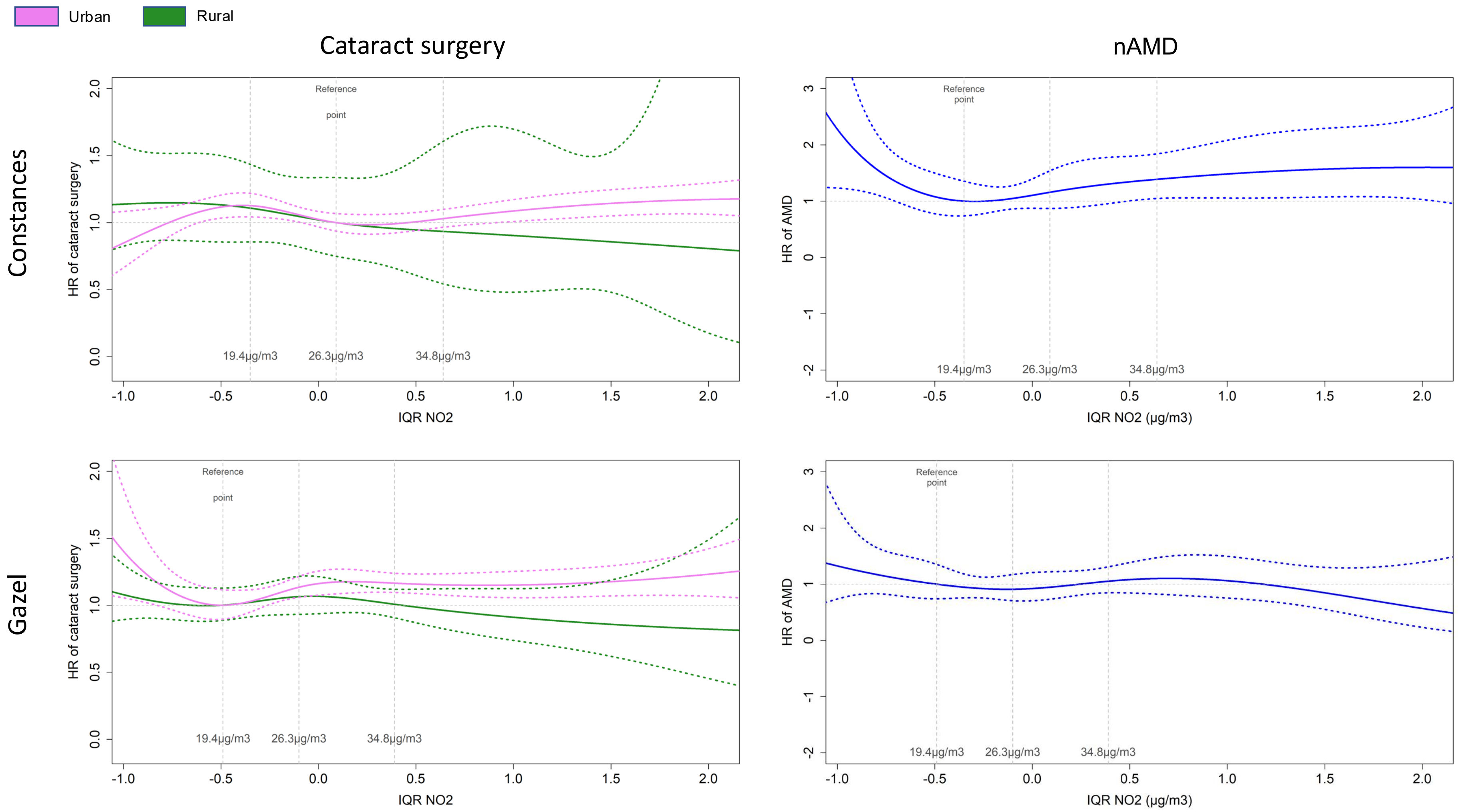

Figure S5. Evolution of cataract surgery and neoascular age-related macular degeneration (nAMD) risk according to $PM_{2.5}$ concentration estimated using splines

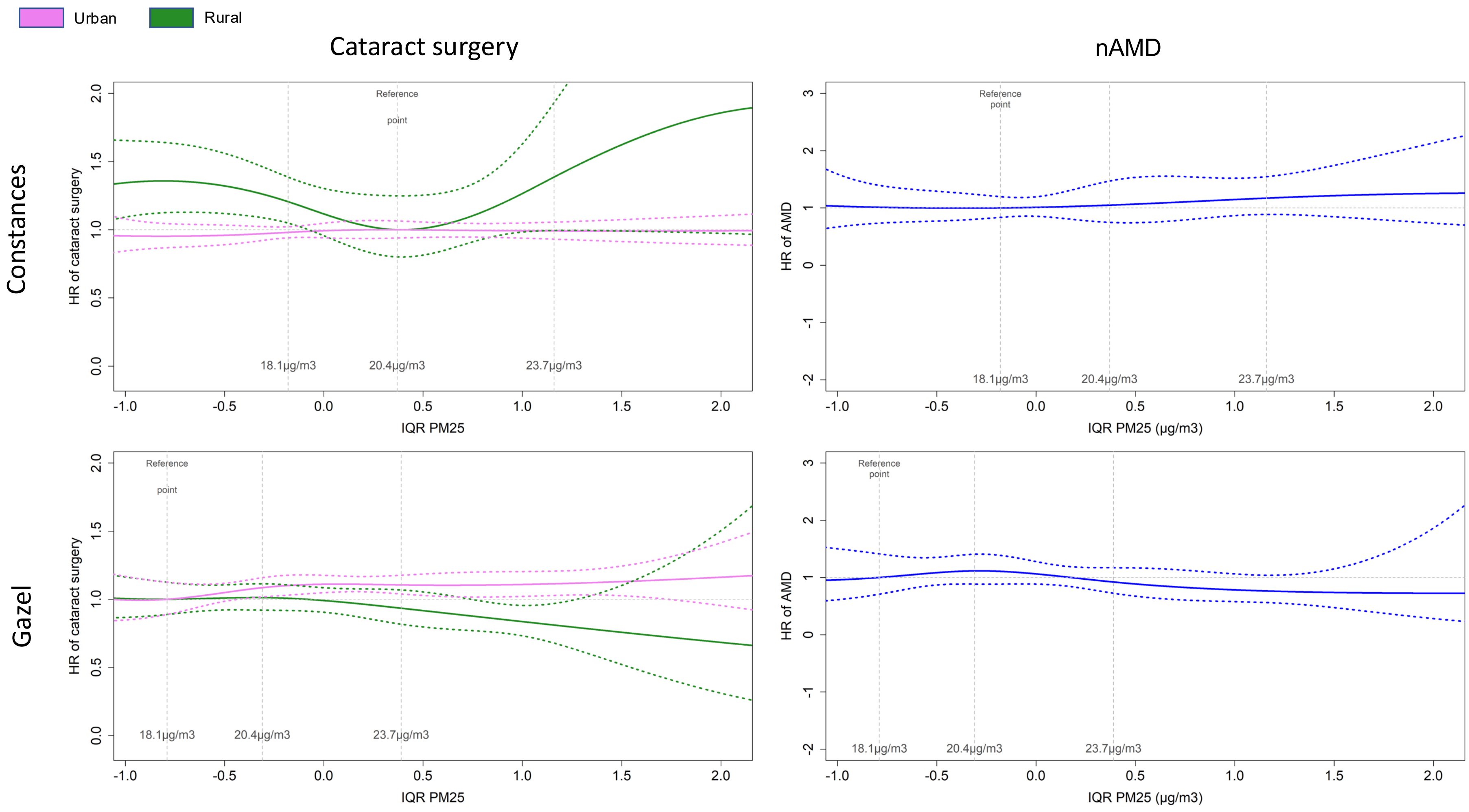

Figure S6. Evolution of cataract surgery and neoascular age-related macular degeneration (nAMD) risk according to BC concentration estimated using splines

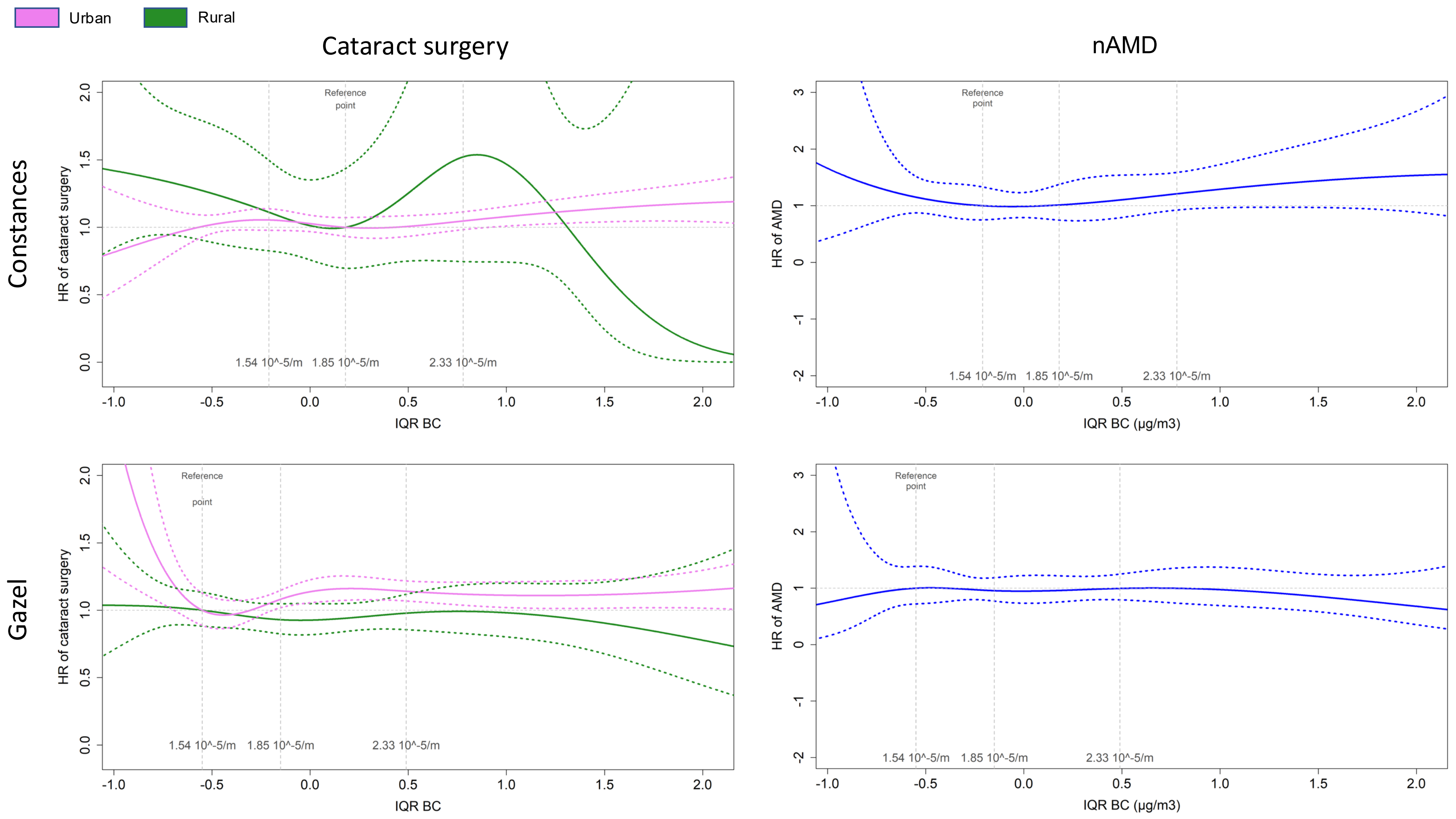

Sensitivity analysis

Sensitivity Analysis 1: Extended Adjustment Including Outcome Risk Factors

Models were additionally adjusted for established risk factors of the outcome, beyond the confounders included in the primary analysis, in order to assess the robustness of the observed associations.

Figure S7. Forest plot of associations between air pollution exposure and risk of cataract (Model 2)

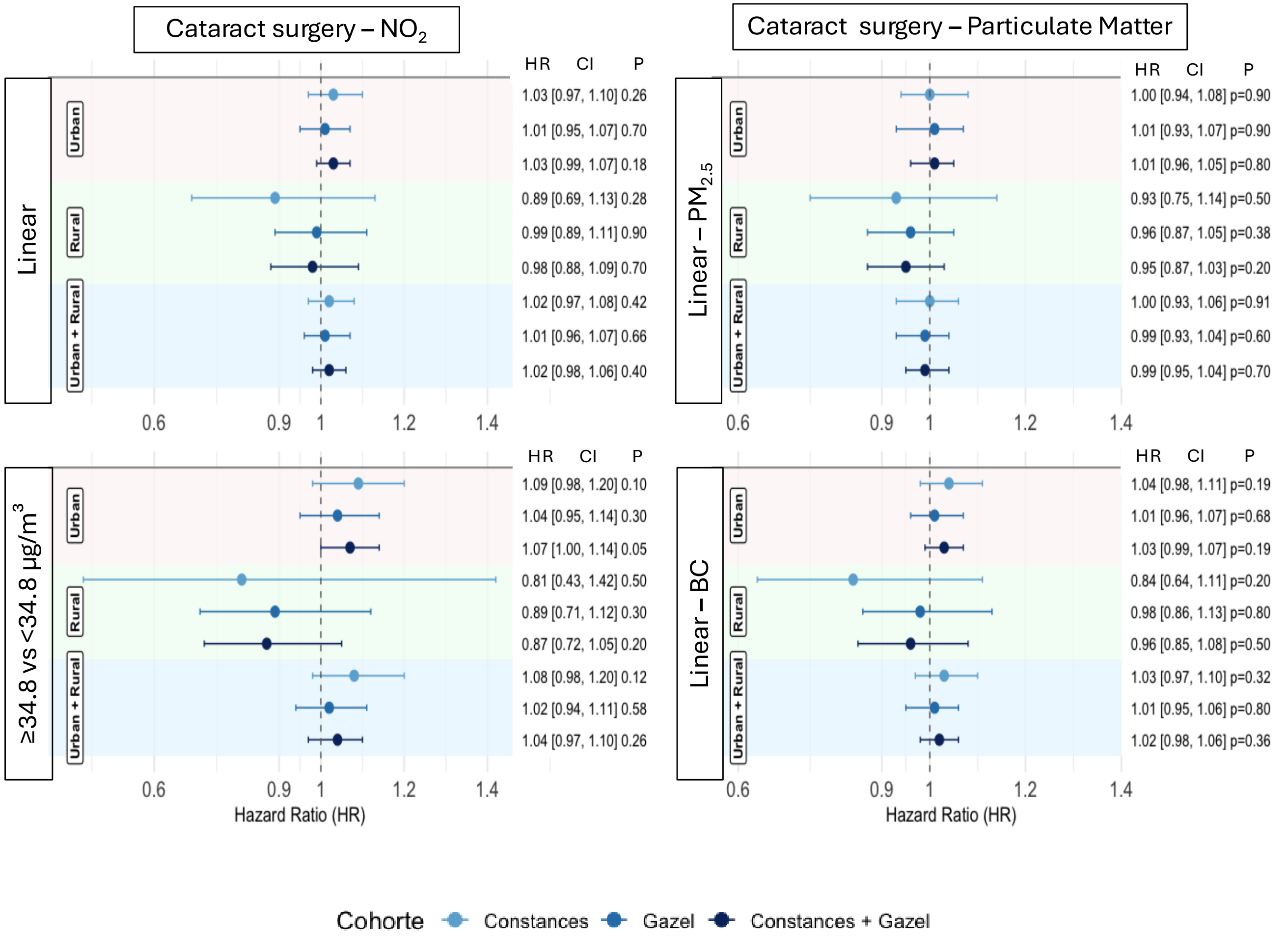

Model 2 was adjusted for age, sex, educational level, area of residence, smoking status, body mass index (BMI) categories, diabetes, hypertension, and corticosteroid use. For analyses combining the Constances and Gazel cohorts, models were additionally adjusted for cohort.

Figure S8. Forest plot of associations between air pollution exposure and risk of nAMD (Model 2)

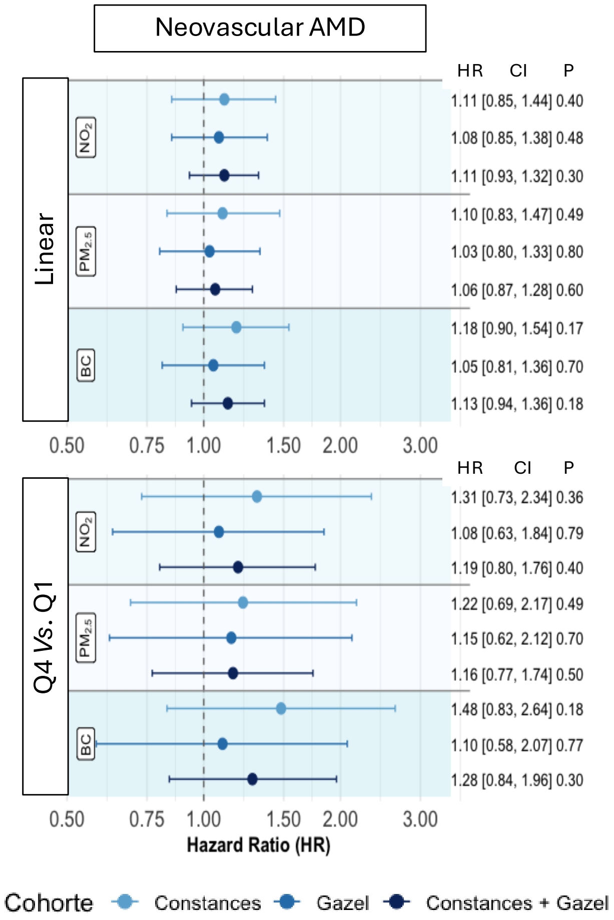

Model 2 was adjusted for age, sex, educational level, area of residence, smoking status, MEDI-LITE score, and hypertension. For analyses pooling the Constances and Gazel cohorts, models were additionally adjusted for cohort.

Sensitivity Analysis 2: Restriction to Participants with High Exposure Data Completeness

Results after excluding participants with less than 80% completeness of pollution data over the 10-year exposure period

Table S2. Baseline characteristics of the study population

Characteristic	**Overall** N = 35,862 N (%)	**Constances** N = 17,951 N (%)	**Gazel** N = 17,911 N (%)	**Overall** **Cataract surgery,** N = 5,501 N (%)	**Overall** **nAMD, N = 265** N (%)
**Women**	13,830(39%)	8,888(50%)	4,942(28%)	2,146(39%)	96(36)
**Age** (Mean (SD))Min-Max	60.17(4.59) 47.16 - 71.00	58.17(4.68) 47.16 - 71.00	62.18(3.49) 54.00 - 68.00	62.52(3.88) 47.67 - 71.00	62.79(3.91) 50.17 - 70.84
**Education level**					
low	5,458(16%)	1,658(9.4%)	3,800(22%)	1,011(19%)	53(20%)
intermediate	17,129(49%)	6,688(38%)	10,441(60%)	2,662(49%)	132(51)
high	12,145(35%)	9,253(52%)	2,892(16%)	1,635(30%)	72(28%)
other	454(1.3%)	49(0.3%)	405(2.3%)	79(1.5%)	2(0.8%)
Unknown	676	303	373	114	6
**Residence area**					
Rural	8,405(27%)	3,291(18%)	5,114(38)	1,315(28%)	79(34)
Urban	23,041(73%)	14,653(82%)	8,388(62)	3,367(72%)	154(66%)
Unknown	4,416	7	4,409	819	32
**Smoking**					
Never smoker	22,319(66%)	7,679(46%)	14,640(85)	3,627(70%)	169(68)
<20 pack/years	8,672(26%)	6,409(39%)	2,263(13)	1,164(22%)	50(20)
>=20 pack/years	2,665(7.9%)	2,442(15%)	223(1)	386(8%)	29(12%)
Unknown	2,206	1,421	785	324	17

**BMI**					
[0,25)	15,426(46%)	8,817(50%)	6,609(42)	2,209(44%)	103(42)
[25,30)	13,549(41%)	6,570(37%)	6,979(44)	2,107(42%)	105(43%)
≥30	4,458(13%)	2,325(13%)	2,133(14)	754(15%)	37(15)
Unknown	2,429	239	2,19	431	20
**Mediterranean diet adherence** Median(IQR) Range	9.60(3.00) 1.20 - 17.40	8.80(3.00) 1.20 - 17.00	10.20(2.70) 4.00 - 17.40	9.80(3.00) 2.90 - 17.30	10.00(3.00) 5.00 - 16.20
**Diabetes**	2,699(7.5%)	1,145(6.4%)	1,554(8.7)	593(11)	26(9.8)
**Hypertension**	3,939(11%)	3,155(18%)	784(4.4)	708(13%)	42(16)
**Cardiovascular diseases**	12,956(51%)	7,524 (42)	5,432(30)	2,267(51%)	113(54%)
**Corticosteroid therapy**	5,161(14%)	2,440(14%)	2,721(15)	944(17%)	41(15)
**NO_2 µg/m³** (Median (IQR))Min-Max)	26.26(16.93) 3.02 - 125.39	24.82(15.25) 4.13 - 125.39	28.04(17.30) 3.02 - 100.39	27.13(17.76) 3.14 - 93.98	27.33(18.15) 5.32 - 74.67
**$PM_{2.5}$ µg/m³** (Median (IQR))Min-Max)	20.39(5.54) 5.01 - 52.43	18.86(4.06) 5.01 - 52.43	21.84(4.71) 5.61 - 33.71	20.87(5.41) 5.93 - 49.67	20.86(4.62) 13.35 - 35.50
**BC 10^{-5}/m** (Median (IQR))Min-Max)	1.85(0.79) 0.51 - 7.06	1.70(0.79) 0.51 - 7.06	1.97(0.77) 1.09 - 5.15	1.91(0.82) 0.89 - 5.94	1.92(0.75) 1.08 - 4.16

SD:standard deviation ; Min-Max : Minimum-maximum ; IQR : Interquartile range ; BMI :body mass index ; NO2 : nitrogen dioxide ; PM2,5 : fine particulate matter ; BC : black carbon

Table S3: Association of Air Pollution with Incidence of cataract Surgery estimated by Cox Models

	Constances			Gazel			Constances + Gazel		
**Characteristic**	**HR[1]**	**95% CI[1]**	**p-value**	**HR[1]**	**95% CI[1]**	**p-value**	**HR[1]**	**95% CI[1]**	**p-value**
**Urban**	N = 14,658; N cataract surgery = 1777			N = 11,060; N cataract surgery = 2095			N = 25,718; N cataract surgery = 3872		
**NO2 (µg/m3)**									
Linear	1.05	0.99, 1.11	0.12	1.03	0.97, 1.09	0.4	1.04	1.00, 1.08	0.083
≥34.8 Vs. <34.8	1.11	1.00, 1.23	0.043	1.06	0.97, 1.16	0.2	1.08	1.01, 1.16	0.024
**PM25 (µg/m3)**									
Linear	1.03	0.96, 1.10	0.5	1.01	0.95, 1.07	0.8	1.02	0.97, 1.07	0.5
**BC (10-5/m)**									
Linear	1.06	0.99, 1.13	0.074	1.02	0.96, 1.08	0.5	1.04	1.00, 1.08	0.074
**Rural**	N = 3,293; N cataract surgery = 383			N = 6,851; N cataract surgery = 1246			N = 10,144; N cataract surgery = 1629		
**NO2 (µg/m3)**									
Linear	0.89	0.70, 1.14	0.4	1.00	0.89, 1.13	>0.9	0.98	0.88, 1.08	0.6
≥34.8 Vs. <34.8	0.81	0.43, 1.52	0.5	0.91	0.71, 1.16	0.4	0.87	0.69, 1.09	0.2
**PM25 (µg/m3)**									
Linear	0.93	0.76, 1.15	0.5	0.95	0.87, 1.04	0.2	0.94	0.87, 1.03	0.2
**BC (10-5/m)**									
Linear	0.85	0.64, 1.13	0.3	1.0	0.87, 1.14	>0.9	0.96	0.86, 1.08	0.5
**Urban + rural**	N = 17,951; N cataract surgery = 2160			N = 17,911 ; N cataract surgery =3341			N = 35,862; N cataract surgery =5501		
**NO2 (µg/m3)**									
Linear	1.04	0.98, 1.10	0.2	1.02	0.97, 1.07	0.5	1.03	0.99, 1.07	0.2
≥34.8 Vs. <34.8	1.10	1.00, 1.22	0.060	1.03	0.95, 1.12	0.5	1.06	1.00, 1.13	0.066
**PM25 (µg/m3)**									
Linear	1.02	0.95, 1.08	0.6	0.99	0.94, 1.04	0.7	1.00	0.96, 1.04	>0.9
**BC (10-5/m)**									
Linear	1.05	0.99, 1.11	0.14	1.02	0.96, 1.07	0.6	1.03	0.99, 1.07	0.2

HR = Hazard Ratio, CI = Confidence Interval

Model 1 adjusted for age, sex and education level after multiple imputations of the covariates; and for study (Constances/Gazel) in the overall analyses

IQR Constances : NO_2 : 15.38µg/m3 ; $PM_{2.5}$: 4.13µg/m3 ; BC : 0.80 10-5/m

IQR Gazel : NO_2 : 17.30µg/m3 ; $PM_{2.5}$: 4.71µg/m3 ; BC : 0.77 10-5/m

IQR Overall : NO_2 : 16.99µg/m3 ; $PM_{2.5}$: 5.55µg/m3 ; BC : 0.80 10-5/m

Table S4: Association of Air Pollution with Incidence of Neovascular AMD Estimated by Cox Models

	Constances			Gazel			Constances + Gazel		
**Characteristics**	**HR[1]**	**95% CI[1]**	**p-value**	**HR[1]**	**95% CI[1]**	**p-value**	**HR[1]**	**95% CI[1]**	**p-value**
**Urban**	N=14,658; N nAMD = 82			N=11,060; N nAMD = 89			N=25,718; N nAMD = 171		
**NO_2 (µg/m3)**									
Linear	1.17	0.89, 1.53	0.3	1.04	0.79, 1.37	0.8	1.10	0.91, 1.34	0.3
≥34.8 Vs. <34.8	1.30	0.81, 2.09	0.3	0.97	0.63, 1.50	0.9	1.11	0.81, 1.53	0.5
**$PM_{2.5}$ (µg/m3)**									
Linear	1.17	0.87, 1.58	0.3	1.01	0.73, 1.40	>0.9	1.10	0.88, 1.36	0.4
**BC (10-5/m)**									
Linear	1.25	0.95, 1.65	0.12	1.03	0.78, 1.37	0.8	1.14	0.94, 1.39	0.2
**Urban + rural**	N = 17,951; N nAMD = 106			N = 17,911 ; N nAMD =159			N = 35,862 ; N nAMD =265		
**NO_2 (µg/m3)**									
Linear	1.14	0.88, 1.49	0.3	1.08	0.85, 1.38	0.5	1.11	0.93, 1.32	0.3
≥34.8 Vs. <34.8	1.38	0.87, 2.17	0.2	1.02	0.69, 1.51	0.9	1.16	0.86, 1.56	0.3
**PM2.5 (µg/m3)**									
Linear	1.13	0.85, 1.50	0.4	1.02	0.79, 1.32	0.9	1.07	0.88, 1.29	0.5
**BC (10-5/m)**									
Linear	1.23	0.94, 1.61	0.14	1.05	0.81, 1.36	0.7	1.13	0.94, 1.36	0.2
Quartiles									
Q1 : <1.54	-	-		-	-		-	-	
Q2: [1.54,1.85)	1.10	0.61, 1.98	0.7	1.03	0.60, 1.78	>0.9	1.07	0.73, 1.56	0.7
Q3: [1.85,2.33)	1.36	0.75, 2.47	0.3	1.38	0.79, 2.43	0.3	1.43	0.97, 2.10	0.073
Q4: ≥2.33	1.63	0.91, 2.92	0.10	1.09	0.58, 2.05	0.8	1.32	0.86, 2.01	0.2

HR = Hazard Ratio, CI = Confidence Interval

Model 1 adjusted for age, sex and education level after multiple imputations of the covariates; and for study (Constances/Gazel) in the overall analyses

IQR Constances : NO_2 : 15.38µg/m3 ; $PM_{2.5}$: 4.13µg/m3 ; BC : 0.80 10-5/m

IQR Gazel : NO_2 : 17.30µg/m3 ; $PM_{2.5}$: 4.71µg/m3 ; BC : 0.77 10-5/m

IQR Overall : NO_2 : 16.99µg/m3 ; $PM_{2.5}$: 5.55µg/m3 ; BC : 0.80 10-5/m

Table S3: Association of Air Pollution with Incidence of cataract Surgery estimated by Cox Models

	Constances			Gazel			Constances + Gazel		
**Characteristic**	**HR[1]**	**95% CI[1]**	**p-value**	**HR[1]**	**95% CI[1]**	**p-value**	**HR[1]**	**95% CI[1]**	**p-value**
**Urban**	N = 14,658; N cataract surgery = 1777			N = 11,060; N cataract surgery = 2095			N = 25,718; N cataract surgery = 3872		
**NO2 (µg/m3)**									
Linear	1.05	0.99, 1.11	0.12	1.03	0.97, 1.09	0.4	1.04	1.00, 1.08	0.083
≥34.8 Vs. <34.8	1.11	1.00, 1.23	0.043	1.06	0.97, 1.16	0.2	1.08	1.01, 1.16	0.024
**PM25 (µg/m3)**									
Linear	1.03	0.96, 1.10	0.5	1.01	0.95, 1.07	0.8	1.02	0.97, 1.07	0.5
**BC (10-5/m)**									
Linear	1.06	0.99, 1.13	0.074	1.02	0.96, 1.08	0.5	1.04	1.00, 1.08	0.074
**Rural**	N = 3,293; N cataract surgery = 383			N = 6,851; N cataract surgery = 1246			N = 10,144; N cataract surgery = 1629		
**NO2 (µg/m3)**									
Linear	0.89	0.70, 1.14	0.4	1.00	0.89, 1.13	>0.9	0.98	0.88, 1.08	0.6
≥34.8 Vs. <34.8	0.81	0.43, 1.52	0.5	0.91	0.71, 1.16	0.4	0.87	0.69, 1.09	0.2
**PM25 (µg/m3)**									
Linear	0.93	0.76, 1.15	0.5	0.95	0.87, 1.04	0.2	0.94	0.87, 1.03	0.2
**BC (10-5/m)**									
Linear	0.85	0.64, 1.13	0.3	1.0	0.87, 1.14	>0.9	0.96	0.86, 1.08	0.5
**Urban + rural**	N = 17,951; N cataract surgery = 2160			N = 17,911 ; N cataract surgery =3341			N = 35,862; N cataract surgery =5501		
**NO2 (µg/m3)**									
Linear	1.04	0.98, 1.10	0.2	1.02	0.97, 1.07	0.5	1.03	0.99, 1.07	0.2
≥34.8 Vs. <34.8	1.10	1.00, 1.22	0.060	1.03	0.95, 1.12	0.5	1.06	1.00, 1.13	0.066
**PM25 (µg/m3)**									
Linear	1.02	0.95, 1.08	0.6	0.99	0.94, 1.04	0.7	1.00	0.96, 1.04	>0.9
**BC (10-5/m)**									
Linear	1.05	0.99, 1.11	0.14	1.02	0.96, 1.07	0.6	1.03	0.99, 1.07	0.2

HR = Hazard Ratio, CI = Confidence Interval

Model 1 adjusted for age, sex and education level after multiple imputations of the covariates; and for study (Constances/Gazel) in the overall analyses

IQR Constances : NO_2 : 15.38µg/m3 ; $PM_{2.5}$: 4.13µg/m3 ; BC : 0.80 10-5/m

IQR Gazel : NO_2 : 17.30µg/m3 ; $PM_{2.5}$: 4.71µg/m3 ; BC : 0.77 10-5/m

IQR Overall : NO_2 : 16.99µg/m3 ; $PM_{2.5}$: 5.55µg/m3 ; BC : 0.80 10-5/m
